# Fear of mating out (FOMO): voyeurism does not increase mating propensity in fruit flies

**DOI:** 10.1038/s41598-024-83465-6

**Published:** 2024-12-30

**Authors:** Regina Vega-Trejo, Krish Sanghvi, Biliana Todorova, Irem Sepil, Eleanor Bath

**Affiliations:** Department of Biology, University of Oxford, 11a Mansfield Road, Oxford, OX1 3SZ UK

**Keywords:** Mate choice, Fruit flies, Behavioural plasticity, Social cues, Evolution, Sexual selection

## Abstract

Mate availability and social information can influence mating behaviour in both males and females. Social information obtained from conspecifics can influence mate choice, particularly shown by studies on mate choice copying. However, the role of directly observing conspecific mating on mating behaviour has been less explored. As such, whether conspecifics are copulating or not could inform ‘observers’ about the availability of mates, or even stimulate observers to mate themselves. Using *Drosophila melanogaster*, we tested whether exposure to the visual cue of a mating pair would increase the mating propensity of an observer pair (i.e., voyeur). We followed a factorial design where a male-female pair (voyeur flies) were placed together with or without visual access to another pair of flies (who were either mating or not mating). We found no evidence that mating latency or duration of mating were affected by whether voyeurs had visual access to a mating or non-mating pair. These results could be due to biological factors (e.g., use of other non-visual cues by flies to acquire information related to sex), or methodological limitations of our study (e.g., flies unable to watch other pairs). Generally, our results suggest that fruit flies do not use visual cues from conspecifics mating to adjust their own mating latency or mating duration.

## Introduction

Animals are often able to gather information about their environment from conspecifics and can use this information to make decisions that influence their social and sexual behaviour^1–3^. In some cases, behavioural interactions with conspecifics can even provide individuals with more accurate and reliable information about mating decisions, than the information acquired by observers themselves^1,3,4^. Social information is especially important in situations where individuals are naïve and lack prior information, or when information collected by individuals is ambiguous^5–7^. For example, virgin females across taxa are more likely to copy mate choices of other females compared to mated females, because virgins lack sexual experience^8^. Young female guppies (*Poecilia reticulata*) tend to choose the same males preferred by older females, a behaviour that is likely associated with reducing the risk of predation and maximisation of foraging time^9^. Social information can be especially crucial when personal information is difficult or costly to acquire^10–12^. For instance, when individuals sample mates sequentially^13^, or when experience with familiar phenotypes can influence mating decisions^14^. Research on socially influenced (non-independent) mating behaviour has mainly focused on mate choice copying^8,15^, which refers to a form of social learning where an individual’s mate choice is influenced by the mate choices of other individuals. These experiments focus on testing whether individuals can use social information by choosing or rejecting a similar individual after observing another individual’s choice^16^. For example, female fruit flies (*Drosophila melanogaster*) that observe coloured males (i.e. covered in either pink or green powder), prefer to copulate with the male of the colour they have previously observed mating^17^.

Processes and cues other than mate choice copying might be involved in affecting social and mating behaviour of conspecifics. An example is the audience effect, where the presence of a ‘voyeur’ individual can influence the behaviour of individuals being observed^18^. In male Atlantic mollies (*Poecilia mexicana)*, focal males who are watched by a potential rival male prefer a previously non-preferred female, compared to focal males not being watched by potential rivals^19^. Similarly, an individual can alter its own mating behaviour when observing conspecifics interact (eavesdropping^20^). For example, sexually experienced female Japanese quails that observe males engage in aggressive encounters prefer to associate with less aggressive males^21^. However, other social cues, such as whether conspecifics are mating or not, might be important but have not been broadly assayed before. Social information acquired by watching conspecifics copulate, could inform voyeurs about mate availability/limitation, or suitability of environmental conditions for mating. For instance, there is evidence for shorter latencies to mate in male goats and bulls when they observe conspecifics mating^22^. As such, observing copulation may temporarily heighten the female’s sexual receptivity towards all males^10^.

Here we use fruit flies (*Drosophila melanogaster)* to study whether mating propensity, mating latency, or mating duration is affected by watching others mate. We aim to assess whether individuals can acquire social cues about mate availability or environmental suitability by watching conspecifics mate and, in turn, use these cues to decide whether to mate with a potential partner. Fruit flies are an ideal system as they use an array of visual cues during courtship and prior to mating^23–26^, as well as auditory, gustatory, olfactory cues^27,28^, and substrate-borne vibrations^29,30^. Both males and females can be choosy due to gamete limitation in both sexes^31–33^. Males show elaborate and costly courtship, and both males and females can discriminate mated from virgin individuals^17,33–36^. Females have been shown to prefer virgin males^33^, likely to avoid mating with sperm-depleted, non-virgin males^37,38^. Similarly, males prefer virgin females, likely to avoid sperm competition^39^. Additionally, no-choice experiments, in which only one male and one female interact, still indicate preference of the female and the male, as longer latencies are associated with lower receptivity or higher selectivity, and have been demonstrated consistently in previous studies^40–42^. Moreover, fruit flies have been the focus of mate choice copying studies in invertebrates (e.g. ^17,36^) suggesting that they are able to use social cues to inform mating decisions and discriminate among potential mates, even when observing other individuals with a transparent partition in between^33,43–45^.

To test whether a male and female in a pair changed their propensity to mate and mating duration if provided with visual access to a mating pair of conspecifics, we gave a focal pair of flies (hereafter, ‘voyeur pair’) either visual (no barrier), or no visual (with a barrier) access to another pair of flies (hereafter, ‘demonstration pair’). This demonstration pair was either copulating, or not, when the voyeur male and female flies were put together. We either placed males and females in voyeur pairs at the same time as the demonstration pair, or when the demonstration pair had just started to copulate. This was done to control for the effect of similar mating latencies between voyeur and demonstration pairs, driven by the fact that pairs were introduced in vials at the same time. We then calculated the lag between the start of mating between the voyeur and the demonstration pair, as well as the mating duration and latency of the voyeur pair and compared these between our four treatments. We focused on voyeurs’ likelihood to mate using mating latency, as this reflects choosiness and receptivity to mating^46,47^; and mating duration, as this is also known to be under female control and reflects female mating experience and can influence the quantity of the ejaculate transferred^47^, hence paternity of the male^48^. The lag between the two vials in their mating latency within a dyad was used to quantify the effect of voyeurism on a continuous numeric scale rather than only as a binary one (i.e. mated/not mated). If watching conspecifics mate stimulates voyeur flies to mate, we predicted that the lag in copulation start times between voyeur and demonstration pairs would be shortest in the treatment with no barrier and when flies were placed at different times (H1). This is because introducing pairs of flies (i.e. dyads) at different times would allow voyeur pairs to have visual access to mating pairs as soon as voyeur pairs were put together. However, if only the time since introduction of a pair in a vial, but not their use of visual cues from mating conspecifics, affects their mating behaviour, we predicted that pairs of flies that were placed in a vial at the same time would have a shorter lag, but visual access to another pair of flies would not affect lag between pair vials (H2). Finally, if the presence of other flies (whether or not they were mating) stimulated mating in voyeur pairs, we predicted pairs of voyeur flies to copulate faster when provided visual access, irrespective of whether they were placed at similar or different times to the demonstration pair (H3).

## Methods

### Stock and fly maintenance

We used 608 individuals from a lab-adapted, outbred Dahomey wild-type stock. This stock was originally collected in Benin, Africa, in 1970 and flies have been maintained since in large, outbred population cages with overlapping generations^49^. Eggs were collected from the population cage and raised at standardized larval density^50^. Experimental flies were collected as virgins within seven hours of emergence using ice anaesthesia. Flies were kept at 25 °C on a 12:12 light-dark cycle and fed Lewis medium supplemented with *ad libitum* live yeast. Fly manipulations were performed by gentle aspiration. All individuals were virgins. Females were kept in individual vials, while males were held in single-sex groups (10 males per vial), as previous exposure to rivals increases mating duration in males^51^. This was done for five days prior to the experiments.

## Experimental design

To test whether flies changed their propensity to mate and mating duration in the presence or absence of mating or non-mating conspecifics, we assigned experimental flies at random to one of four treatments. Each treatment had two plastic transparent vials (henceforth called a dyad), with each vial consisting of a male and a female who were allowed to freely mate. Vials were 11 × 2.3 cm (length× width), though only 3.5 cm of the vials’ length was available to the flies.

**Fig. 1:**
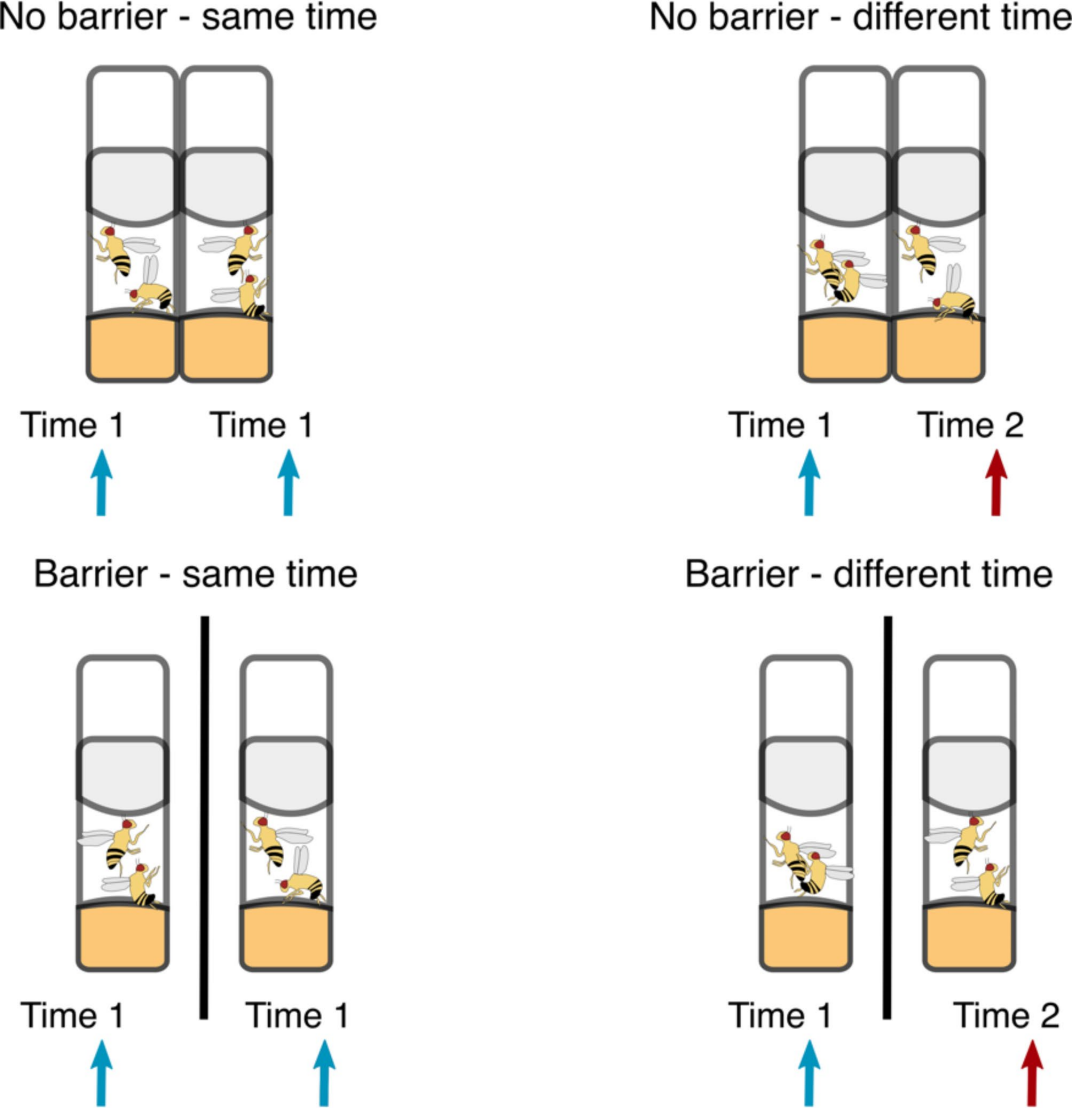
Each treatment consisted of two plastic transparent vials in which a virgin male and a female were placed together. Flies were either given visual access (no barrier – vials placed adjacent to each other) or no visual access (barrier- vials separated by a cardboard partition) to another pair of flies. Moreover, flies in the second vial were either placed at the same time as the flies in the first vial (same time), or alternatively only when the flies in the first vial started copulating (different time). Flies were given about a third of the space in the vial to stimulate mating (3.5 cm in length and 2.3 cm in width).

In our first treatment, a pair of virgin flies was placed into each transparent food vial in the dyad at the same time and allowed free visual access to the other, adjacent vial (no barrier – same time; Fig. 1). In our second treatment, one male and one female were placed into a vial to mate (demonstration pair), and only after flies in this vial started to mate, a second pair of flies (i.e., one male and one female- voyeur pair) were placed into a vial adjacent to the first pair. These voyeur pairs had immediate visual access to mating flies. The first pair in this treatment did not have access to watching another pair of flies before they started copulation, but the second pair had visual access to a mating pair of flies as soon as males and females in the second pair were put together (no barrier – different time; Fig. 1). In these treatments, vials were placed adjacent (touching) to each other to provide visual cues. In the third treatment, one male and one female were placed into both vials in a dyad at the same time. However, vials in this treatment were divided by a solid partition to prevent any visual access between the two paired vials (barrier – same time; Fig. 1). Finally, in our fourth treatment, one male and one female were placed in a vial, and as soon they started copulating, one male and one female were placed into a different vial. In this treatment, the vials in the dyad were separated by a solid partition to prevent visual access (barrier – different time; Fig. 1). In these treatments, vials were placed as close together as possible, albeit separated by a cardboard partition. For each vial we recorded mating latency and mating duration. All pairs in a vial were given four hours to mate, with three experimenters observing the matings, and matings were recorded to the minute. We further recorded the time elapsed between the start of copulation in the first and second vials for pairs in each given dyad (henceforth called lag). Overall, we had 304 male and female mating pairs (i.e., 38 dyads per treatment), conducted over four days (replicates).

### Data analysis

We used R v. 4.1.2^52^ for all analyses. All linear models were checked for model assumptions of normality and homoscedasticity of residuals using the *stats* package. All generalised linear models were checked for overdispersion using the *DHARMa* package^53^.

We first tested whether the difference in time between the start of copulation of flies in a dyad (i.e., lag) was affected by whether a pair of flies in a vial had visual access to mating or non-mating conspecifics in another vial. We calculated lag by subtracting the time when the first mating pair in a vial started copulating from the time when the second pair in a different vial started copulating. Note that this difference was considered using absolute values (i.e., no negative values) as for ‘barrier – different time’ and ‘no barrier – different time’ treatments, the first mating pair was always the pair that was placed in the vial first. However, in the ‘barrier – same time’ and ‘no barrier – same time’ pairs, it could have been either pair in the dyad. We log transformed the lag values to fulfil the model assumptions of normality and homoscedasticity of residuals.

We then tested whether this lag (dependent variable) was influenced by the two-way interaction between the presence or absence of a barrier and whether a pair of flies were introduced at the same or different time in a dyad, as fixed effects. Day of the experiment was also included as a fixed effect.

Finally, we tested whether barrier presence or time of introduction in vials affected mating latency and mating duration of pairs in a vial, in two separate models, with duration and latency as the dependent variables, respectively. For both models, we created a generalised linear mixed model using the *glmmTMB* package^54^. We included barrier (presence vs. absence), time of introduction (same time vs. different time), and their interaction as fixed effects, as well as day of the experiment. We also included pair identity as a random factor to account for the non-independence of the dyad. Note that this random effect was not included in the lag model, as each dyad only had a single value.

## Results

**Fig. 2:**
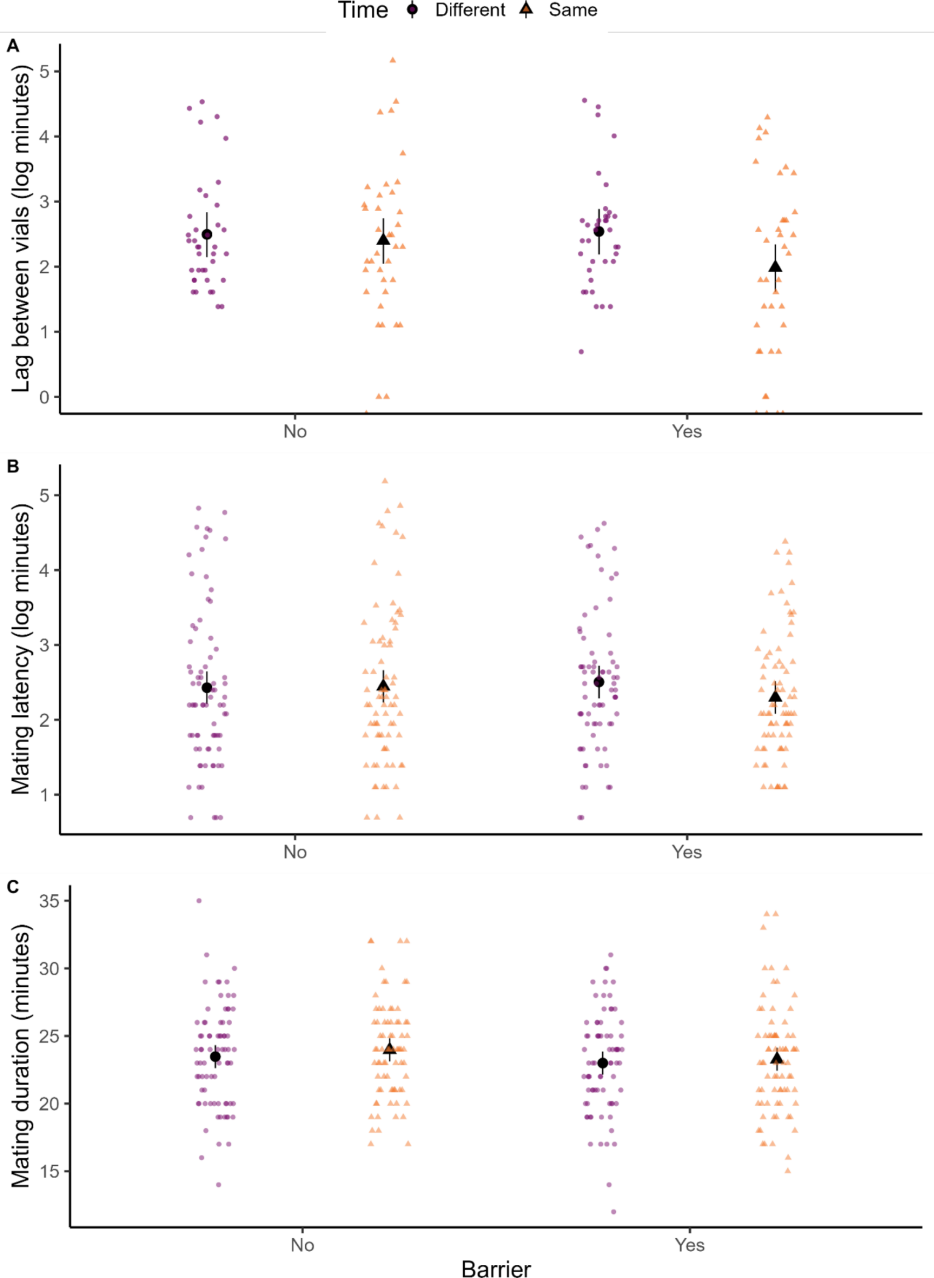
Effect of barrier (No - visual access to another mating pair vs. Yes - no visual access) and time of introduction of flies (orange triangles - mating pair placed at the same or purple circles - different time in the vials of a dyad) on the lag between vials to start copulation (**A**), mating latency of flies in a vial (**B**), and their mating duration (**C**). Black points and error bars represent means and 95% CI from model outputs. Raw data points shown in colour which for “lag” are one point per dyad, and for latency and duration, are two per dyad.

Overall, 303 out of 304 mating pairs copulated. The lag between vials in a dyad for flies to start copulating was not affected by the interaction between barrier and time of introduction of flies (t = -1.279, *P* = 0.203; Fig. 2A), nor by main-effects of barrier (t = -0.181, *P* = 0.857) or time of introduction of flies (t = -0.385, *P* = 0.701). Similarly, mating latency was not affected by the interaction between barrier and time of introduction of flies (z = -1.159, *P* = 0.309; Fig. 2B), or a main effect of barrier (z = -0.489, *P* = 0.625), or time of introduction of flies (z = 0.111, *P* = 0.911). Finally, mating duration was not affected by the interaction between barrier and time of introduction of flies (z = -0.26, *P* = 0.798; Fig. 2C), nor barrier (z = -0.78, *P* = 0.435), or time of introduction of flies (z = 0.82, *P* = 0.412).

## Discussion

Whether individuals decide to mate with an available potential partner can be influenced by various socially acquired cues^11,55^. Here, we tested if observing a conspecific pair (mating or not), would make voyeur pairs more likely to mate themselves. Contrary to predictions, we did not find any evidence for mating behaviour in fruit flies to be influenced by the presence or mating behaviour of another conspecific pair in proximity. We discuss the role of several biological and methodological factors that might explain this lack of effect.

Observing other pairs mate can provide cues for mate availability and limitation. Even when conspecifics are not mating, visual cues to their presence or absence may inform observers about group size, numbers of competitors^56–58^, or sex ratio^43^. Such social cues regarding mate availability can make individuals less choosy^59^, leading individuals to mate with whatever potential partners are available. For instance, in two-spotted gobies, females become less choosy toward preferring larger males by the end of the breeding season, when the availability of males decreases, compared to the start of the season^60^. Our experiment, however, did not find any evidence in support of such plasticity in mating behaviour. This lack of effect can be explained in numerous ways. First, our study consisted of a no-choice experiment, where neither males or females were allowed to choose between different individuals. Their choice was limited to when to start mating and for how long, traits which may have not suitable for testing social information driven choice.

Secondly, the lack of results might indicate that flies are unable to use the visual cues we provided to make inferences about potentially available mates. It is possible that direct interaction between individuals is needed to elicit a response. For instance, *D. melanogaster* males with no social experience that are housed with females as an indicator of female abundance, exhibit higher courtship effort compared to males who are either housed with other males as an indication of potential competition, or males housed alone^61^. In contrast, exposure to rival males can serve as a cue for sperm competition. In line with this, male fruit flies mate for longer when exposed to rivals prior to mating, but reduce mating duration when rivals are present during a mating event^51^.

Whether males initiate courtship and whether females choose to mate is likely to be affected by a range of cues, in addition to the visual cues we provided. Male fruit flies use sight, smell, and taste to detect females, but do not need all senses simultaneously to be able to do so^62^. It is therefore possible that in our experiment, visual cues of copulating pairs might not have been sufficiently salient to influence the propensity for mating of voyeur pairs, or that pairs in other vials were not detected at all. This could be because voyeur pairs might not have detected mating behaviour of the demonstration pairs, without being exposed to their courtship behaviour^63^, pheromones^64^, chemical cues^65^ or a combination of multiple cues^28^. Future studies could provide flies with other types of cues about conspecific mating, and test whether this influences a voyeur pair’s mating propensity.

Our design was based on the use of visual cues because these cues have been shown to play an important role in courtship behaviour in flies^23–25^ and in some cases affect mate-choice copying. Several studies have indeed shown that focal *Drosophila* females prefer the male phenotype chosen by another female when they are provided with visual cues, including different male wing shapes^44^ or artificially coloured males as evidenced by meta-analyses^8,15,]^and other individual studies^17,45,]^, but see^66,67^. Yet, the focus of studies on non-independent mate choice remain centred on mate choice copying^68^. Based on these studies, we assumed that flies would be able to observe individuals in adjacent vials. However, we used plastic and not glass vials, which may have affected their ability to detect other flies. The fly strain used in our study also differs from the strain used in other studies which might have impacted the results. Moreover, as indicated above, females in our set up were not exposed to multiple males to choose from, nor multiple cues. Hence, our study did not focus on mate choice copying, but rather explored whether observing another individual mating could trigger mating behaviour, which we found no evidence to support.

Watching conspecifics mate might not only indicate mate availability, but also provide cues of other processes. For males, watching another male mate might indicate presence of rivals^69^, which can inform mating decisions in species such as flies where sperm competition is high^70,71^. Thus, whether mating behaviour changes or not in the presence of other individuals might require a more nuanced understanding of the information being obtained by watching others mate. In some instances, voyeurism might decrease mating behaviour, as shown in male guppies who decrease their mating effort when rivals are present^72^. Our results suggest that either males or females are unable to alter their mating behaviour due to cues of increased sperm competition risk, or that positive versus negative pressures on mating behaviour might be balancing each other out.

## Conclusion

We tested whether a pair of potential mates are more willing to copulate when they can watch conspecifics mate. We found no evidence for this. Our lack of significant results could be due to the inability of flies to see the other pair or their inability to acquire social information that influences mating decisions by simply watching other flies mate. It could also be driven by the indifference of flies to change their behavioural decisions despite having access to social information, either because such information is not useful or informative, or multiple cues cancel each other out (such as lower virgin mate availability *versus* higher numbers of available mates). Despite these limitations, our research is crucial for highlighting a less studied aspect of social information use and can inform future studies on such questions.

## Data Availability

Data and code are available at OSF—https://osf.io/fntbr/?view_only=d86dfcd542974009b052819f63d7c135.
